# The Application of Ultrasound in Honey: Antioxidant Activity, Inhibitory Effect on α-amylase and α-glucosidase, and In Vitro Digestibility Assessment

**DOI:** 10.3390/molecules27185825

**Published:** 2022-09-08

**Authors:** Armando Peláez-Acero, Diana Belem Garrido-Islas, Rafael Germán Campos-Montiel, Lucio González-Montiel, Gabriela Medina-Pérez, Lorena Luna-Rodríguez, Uriel González-Lemus, Antonio de Jesús Cenobio-Galindo

**Affiliations:** 1 Instituto de Ciencias Agropecuarias, Universidad Autónoma del Estado de Hidalgo, Av. Rancho Universitario s/n Km. 1., Tulancingo Hidalgo 43600, Mexico; 2 Instituto de Tecnología de los Alimentos, Universidad de la Cañada, Teotitlán de Flores Magón, Oaxaca 68540, Mexico; 3 José Carlos Rodríguez-Figueroa’s Laboratory, Universidad Autónoma Metropolitana, Unidad Iztapalapa, Avenida San Rafael Atlixco 186, Colonia Vicentina, Mexico City 09340, Mexico

**Keywords:** enzyme inhibition, phenols, flavonoids, botanical origin, ultrasound

## Abstract

In the present study, the effects of ultrasound (10, 20, and 30 min) on the bioactive compounds, antioxidant capacity, enzymatic inhibition, and in vitro digestion of six honey extracts from the Oaxaca state, Mexico, were analyzed. Significant differences were found in each honey extract with respect to the ultrasonic treatment applied (*p* < 0.05). In the honey extract P-A1 treated with 20 min of ultrasound, the phenols reached a maximum concentration of 29.91 ± 1.56 mg EQ/100 g, and the flavonoids of 1.92 ± 0.01 mg EQ/100 g; in addition, an inhibition of α-amylase of 37.14 ± 0.09% was noted. There were also differences in the phases of intestinal and gastric digestion, presenting a decrease in phenols (3.92 ± 0.042 mg EQ/100 g), flavonoids (0.61 ± 0.17 mg EAG/100 mg), antioxidant capacity (8.89 ± 0.56 mg EAG/100 mg), and amylase inhibition (9.59 ± 1.38%). The results obtained from this study indicate that, in some honeys, the processing method could increase the concentration of bioactive compounds, the antioxidant capacity, and the enzymatic inhibition; however, when subjected to in vitro digestion, the properties of honey are modified. The results obtained could aid in the development of these compounds for use in traditional medicine as a natural source of bioactive compounds.

## 1. Introduction

Diabetes mellitus is a serious chronic disease, with high levels of prevalence throughout the world, and it occurs when there are high levels of glucose in a person’s blood, because patients with this condition have poor insulin sensitivity and have difficulty lowering their blood glucose level to the normal range after eating [1]. Specifically, type II diabetes is a condition in which long-chain carbohydrates are hydrolyzed by pancreatic α-amylase and α-glucosidase enzymes, followed by glucose uptake in the intestine, resulting in a sudden increase in blood glucose levels [2].

Since ancient times, honey has occupied an important place as a natural sweetener and as an aid in the treatment of various diseases [3], due to the biological activity that it possesses; it has shown antitumor, anti-inflammatory, and antimicrobial effects, among others, but there is still insufficient information on its specific use in metabolic disorders such as diabetes mellitus [4], and the existing evidence suggests that honey has biological components with antidiabetic potential, including compounds with antioxidant activity [5]. Honey contains phenolic compounds, such as flavonoids, which are transferred through the nectar to honey and are responsible for some of the most important characteristics, such as color, flavor, and its functional properties. All these characteristics are unique and specific to each honey—specifically, depending on its floral origin [6]—and these compounds have been studied due to the relationship that exists with the inhibition of α-glucosidase and α-amylase, indicating great potential for their use in various treatments [7]. The inhibition of the enzymes α-amylase and α-glucosidase significantly decreases the digestion and absorption of carbohydrates, thus lowering the postprandial blood glucose level in people with these conditions. Due to the high content of sugar in honey, it is important to extract the bioactive compounds related to the inhibition of these enzymes in honey for later use [8].

The stability and absorption of bioactive compounds can be modified during gastric and intestinal digestion depending on the food matrix and pH, the presence of enzymes, and even the microbiota and other related factors [9]. The absorption of phenolic compounds is considered low, not exceeding plasma concentrations, and attributed at least partially to the chemical structures of the different polyphenols [10]. A technology that has demonstrated its effectiveness for the extraction of bioactive compounds and that has recently been the subject of multiple investigations is ultrasound, because this extraction methodology has the advantage of obtaining better process times, improving the quality of the matrix to which it is applied, reducing chemical risks, and consuming less energy [11]. Peláez-Acero et al. [12] applied ultrasound to various honeys, finding that this treatment helps to reduce the formation of crystals, does not lead to the formation of hydroxymethylfurfural, and also helps to potentiate the antimicrobial effect, due to the better extraction of bioactive compounds. The objective of the present research was to determine the effects of the in vitro digestion of bioactive compounds, from Mexican honeys, with the ability to inhibit α-amylase and α-glucosidase enzymes extracted by ultrasound, thus generating results that could contribute to future alternatives for treatment against metabolic diseases, such as diabetes, which are increasingly prevalent in developing countries, using ingredients endemic to their regions [6].

## 2. Results

### 2.1. Melissopalynological Analysis

The results of the melissopalynological analysis are presented in Table 1. The honeys have varying botanical origins, among which J-A contains a higher percentage from *Macroptilium* (21.30 ± 1.50%), J-A1 contains more pollen from *Trema micrantha* (13.70 ± 0.10%), and P-B contains a greater amount of pollen of the same floral origin: *Brassica campestris* (35.70 ± 0.20%). In the melissopalynological analysis, the similarity in the origins of the pollen in various treatments was indicated, highlighting that *Trema micrantha* was found in the extracts of J-A, J-A1, P-B, P-A1, and A-B, while *Vitis tiliifolia*, being the most prevalent species, showed a presence in J-A1, P-A, P-B, P-A1, and A-B.

### 2.2. Total Phenolic Content

**Figure 1:**
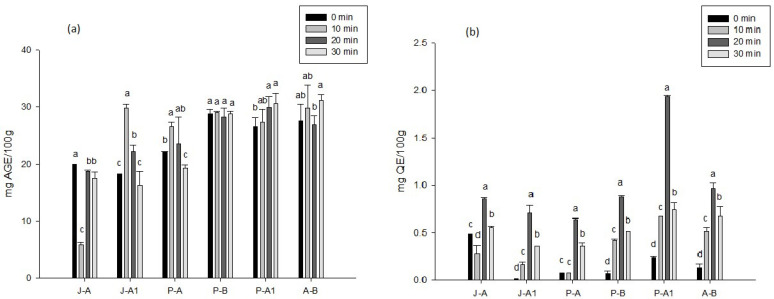
Effects of different ultrasound times (0, 10, 20, 30 min) on the bioactive compounds of different honeys: (**a**) total phenols and (**b**) total flavonoids. The results are expressed as means ± standard deviation. Different letters represent significant differences (*p* < 0.05) between treatments.

The results for the total phenols of the honey extracts under different ultrasound treatments can be seen in Figure 1a. There were significant differences in terms of their processing method in most of the samples (*p* < 0.05), with the exception of P-B, where its content was not affected with respect to the ultrasound time. In J-A1, P-A, and P-A1, the content of total phenols increased at a certain processing time; for example, in J-A1, the content of phenols at 10 min of ultrasound increased from 18.31 ± 0.01 to 29.85 ± 0.58 mg AGE/100 g.

The six types of pollen with the highest frequency in appearance are presented; in general, these grains represent more than 50% of the total pollen quantified.

### 2.3. Total Flavonoids

Regarding the content of total flavonoids (Figure 1b), significant differences (*p* < 0.05) were observed among the different ultrasound processes applied to the honeys, observing that, with the exception of J-A and P-A, all the samples presented an increase with respect to the ultrasound time, highlighting that the greatest increase occurred when 20 min of treatment was applied; P-A1 was the one that presented a greater increase (1.92 ± 0.01 mg QE/100 g), and A-B also presented a significant increase (0.96 ± 0.05 mg QE/100 g). It is possible to note that all samples were affected with 30 min of ultrasound.

### 2.4. Antioxidant Activity by Inhibiting the ABTS Radical

**Figure 2:**
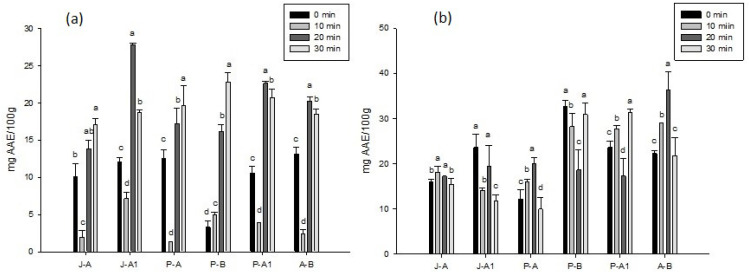
Effects of different ultrasound times (0, 10, 20, 30 min) on the antioxidant activity of different honeys: (**a**) ABTS and (**b**) DPPH. The results are expressed as means ± standard deviation. Different letters represent significant differences (*p* < 0.05) between treatments.

The results of the antioxidant activity achieved through the inhibition of the ABTS radical (Figure 2a) show that there were significant differences (*p* < 0.05) between the treatments with respect to the different times of ultrasound, as well as for the content of bioactive compounds after 20 min. Antioxidant activity increased significantly for all treatments: the highest activity was found in J-A1 at 20 min, with 27.78 ± 0.30 mg AAE/100 g. P-B presented the lowest antioxidant activity at the time 0 (3.26 ± 0.91 mg AAE/100 g); however, at 20 and 30 min, the observed increase was significant (16.17 ± 0.91 and 22.83 ± 1.21 mg AAE/100 g, respectively).

### 2.5. Antioxidant Activity by Inhibition of the DPPH Radical

Figure 2b presents the results for the antioxidant activity achieved through the inhibition of the DPPH radical, where significant differences were found between the treatments (*p* < 0.05) with respect to the applied ultrasound time. The honeys that presented the highest initial antioxidant activity were J-A1 (23.61 ± 2.89 mg AAE/100 g), P-B (32.73 ± 1.31 mg AAE/100 g), and P-A1 (23.61 ± 1.28 mg AAE/100 g), while the ones with the highest activity when subjected to ultrasound with respect to their initial values were P-A (19.96 ± 0.39 mg AAE/100 g), P-A1 (31.36 ± 0.64 mg AAE/100 g), and A-B (36.37 ± 3.86 mg ESA/100 g).

### 2.6. Inhibition of α-amylase

**Figure 3:**
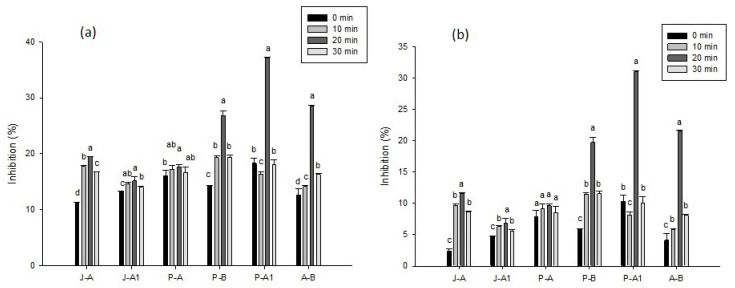
Effects of different ultrasound times (0, 10, 20, 30 min) on the bioactive compounds of different honeys: (**a**) α-amylase and (**b**) α-glucosidase. The results are expressed as means ± standard deviation. Different letters represent significant differences (*p* < 0.05) between treatments.

Figure 3a shows the results for the inhibition of α-amylase when using extracts from honey from the state of Oaxaca, Mexico, indicating that there were significant differences (*p* < 0.05) between the extraction times for each sample and for most treatments, with the exception of P-A, which showed unchanged behavior regardless of the applied treatment. It should be noted that all the samples without treatment presented enzymatic inhibition, and the sample that had the greatest inhibition was P-A1 (18.32 ± 0.91%). The sample that initially presented the least inhibition was J-A (11.15 ± 0.21%), while P-B, P-A1, and A-B showed an increase in inhibition when ultrasonic treatment was applied for 20 min (26.86 ± 0.75, 37.14 ± 0.09, and 28.53 ± 0.1%, respectively).

### 2.7. Inhibition of α-glucosidase

The results for the inhibition of α-glucosidase (Figure 3b) show that there were significant differences (*p* < 0.05) between the various ultrasonic treatments for each sample, indicating increased inhibition, with the exception of P-A. The greatest inhibition occurred at 20 min of ultrasound, in P-B, P-A1, and A-B, where the greatest increase was found (19.70 ± 0.83%, 30.99 ± 0.10%, and 21.54 ± 0.10%, respectively).

### 2.8. In Vitro Digestion

For in vitro digestion, the results are presented for the treatment that was considered the most successful (namely, 20 min) because bioactive compounds, antioxidant activity, and enzyme inhibition all showed an increase in most treatments compared to the other times. Table 2 shows the results for total phenols in the initial, gastric, and intestinal phases, illustrating that the compounds decreased significantly (*p* < 0.05) when they were subjected to the gastric phase; A-B was the treatment that presented a lower decrease in phenolic compounds (4.40 ± 0.02 mg GAE/100 g) with respect to the intestinal phase, and the P-A, P-B, and P-A1 treatments did not present significant differences with respect to the results of the gastric phase.

In terms of total flavonoids (Table 2), the results of the gastric phase followed a behavior similar to that of total phenols, decreasing for most of the samples, with the exception of J-A1 and A-B, which behaved the same initially and after this phase. P-A was the sample type that presented the lowest values after the gastric phase, with 0.10 ± 0.01 mg QE/100 g. It was observed that A-B showed the highest concentrations in all phases (0.97 ± 0.06, 0.86 ± 0.09, and 0.80 ± 0.04 mg QE/100 g, respectively).

For the antioxidant activity achieved through the inhibition of the ABTS radical (Table 2), all the samples suffered a decrease in their activity from the gastric phase to the intestinal phase. J-A1 was the one that showed greater activity after the gastric phase (21.81 ± 1.35 mg ESA/100 g), followed by P-A1 (19.19 ± 0.34 mg ESA/100 g), which also showed greater activity at the end of the test, with 10.41 ± 0.34 mg ESA/100 g. On the contrary, the sample that presented the lowest activity at the end of in vitro digestion was J-A with 7.30 ± 0.68 mg AAE/100 g.

The antioxidant activity results achieved by inhibition of the DPPH radical after in vitro digestion (Table 2) indicated that, after the gastric phase, the highest activity was found in A-B (19.77 ± 0.72 mg AAE/100 g) and J-A1 (18.24 ± 1.45 mg AAE/100 g), noting that J-A, J-A1, P-B, and P-A1 did not present significant differences (*p* > 0.05) from the initial antioxidant activity. At the end of the intestinal phase, P-B was the sample that showed the highest activity (15.16 ± 1.45 mg AAE/100 g) as observed in ABTS, highlighting that this sample also did not present significant differences throughout the digestion, thus being the sample that best preserved the antioxidant activity.

Table 3 shows the results for the inhibition of α-amylase activity, illustrating that there were differences (*p* < 0.05), with a decrease observed in all the treatments between the initial activity and after the gastric phase. The control (acarbose) showed the greatest inhibition: P-A1 was the honey treatment that presented the greatest inhibition, with 19.56 ± 0.29%. For the intestinal phase, differences were observed with respect to the gastric in three samples, with J-A, P-B, and A-B being statistically equal; these latter two presented the greatest enzymatic inhibition at the final level of the test (14.13 ± 2.29% and 13.54 ± 0.21%, respectively), after the control.

The results for the inhibition of α-glucosidase during in vitro digestion can be seen in Table 3. The control (acarbose) showed the greatest inhibition. In terms of α-amylase, all honey treatments decreased during gastric digestion, and A-B was the sample that, at the end of this phase, presented the greatest inhibition (14.50 ± 0.18%), while P-A presented the least activity (7.42 ± 0.35%). During the intestinal phase, all honey treatments suffered a decrease with respect to the previous phase, and P-A1 and A-B were those that completed the test with greater inhibition (9.75 ± 0.04% and 9.62 ± 0.22 %, respectively).

## 3. Discussion

### 3.1. Melissopalynological Analysis

The melissopalynological characterization of honey is essential to define the floral species from which it originates, because, with this information, it is possible to establish quality standards for honey, such as flavor, sugar content, and even the bioactive compounds that it possesses. If the geographical areas of honey production are very close, the geographical identification of the honey is difficult, since its variations are minimal; they are influenced by the climatic conditions and the flowering season [13]. González Sandoval et al. [14] carried out a melissopalynological analysis of honeys from four municipalities in Mexico, mentioning that the families found with the highest prevalence were Convolvulaceae, Asteraceae, Malvaceae, Leguminosae, and Poaceae, and finding that the vast majority were multifloral honeys; they stated that, when there are sufficient sources of pollen in large quantities, bees obtain their food from the plants that are more attractive, without having to compete for limited resources. Meanwhile, as the available resources decrease, they approach other species of plants, diversifying the pollen spectrum.

### 3.2. Bioactive Compounds

Phenolic compounds in honey are of the utmost importance. Hernández-Fuentes et al. [6] determined the content of total phenols in some Mexican honeys, finding that variations in content are mainly due to botanical and geographic origin. Jaafar et al. [15] determined the phenolic content of various multifloral honeys from Lebanon, finding higher values (52.31 ± 9.23 mg/100 g) than those of the honeys analyzed in the present study. Ruiz-Navajas et al. [16] determined the concentrations of phenolic compounds in 11 honeys from Southern Mexico, finding that the values ranged between 51.32 and 134.02 mg/100 g, and mentioning that the content of phenolic compounds could be used as an indicator of the antioxidant capacity for honeys intended to be used as sources of antioxidants in functional foods. Hernandez Fuentes et al. [6] found a wide range of values for total phenols in Mexican honeys, ranging from 18.02 ± 0.49 to 102.77 ± 1.29 mg GAE/100 g, which indicates that these values are highly susceptible to the floral source of the honey. Stojković et al. [17] (2021) showed similar behavior in honey treated with ultrasonic methods, slightly increasing the concentration of phenols in the honey at the beginning but presenting a decrease with respect to greater exposure.

Chaikham et al. [18] subjected honey samples to various ultrasound treatments, mentioning that this method allows better preservation of bioactive compounds, such as flavonoids, compared to conventional thermal methods, and that the increase in flavonoids can be attributed mainly to cell degradation, as the membranes break and the enzymes are released; specifically, in honey, this is due to the disintegration of the pollen grain. Stojković et al. [17], in their study, when evaluating the concentration of total flavonoids, observed that the concentration of flavonoids decreased slightly with respect to ultrasound time. This is similar behavior to that observed by Quintero-Lira et al. [19] (2016), who observed that the ultrasound time increases the concentration of various flavonoids present in honey, such as rutin, quercetin, apigenin, and kaempferol, finding higher concentrations at between 10 and 15 min of sonication.

The decrease in bioactive compounds in the first several minutes can be explained according to the works of Masuda et al. [19] and Dzah et al. [20], who mentioned that ultrasonic irradiation close to 40 kHz favors the formation of unstable bubbles that cause temporary cavitation. These unstable bubbles collapse and generate hydrogen atoms and hydroxyl radicals, resulting in a decrease in bioactive compounds, which can generate antioxidant activity. This phenomenon occurs in the first few minutes. Subsequently, as time passes, the ultrasound allows the accelerated release of the compounds of interest present (phenols, flavonoids, etc.), resulting in an increase [21].

### 3.3. Antioxidant Activity

The behavior of the antioxidant activity observed through the inhibition of the ABTS radical derived from the ultrasound treatment can be attributed to what was reported by Quintero-Lira et al. [22]: that the improvement in the antioxidant activity in honey is due to the increase in the availability of phenolic acids and flavonoids, since the mechanism of action of ultrasound increases the extraction of bioactive compounds. Stojković et al. [17] mentioned that the antioxidant activity of honey can be attributed to various compounds, among which phenolic acids, flavonoids, and some enzymes stand out, and it is known that this has a wide correlation with other attributes, such as the color. Yalçin [23] subjected honey from Turkey to different ultrasound (5, 10, 15, and 20 min) and temperature (30, 45, 60, and 80 °C) conditions, finding that the antioxidant activity values obtained by ABTS were affected mainly by thermal treatment and to a lesser extent by ultrasound; they observed that the values of their samples increased with the increase in the ultrasound time compared to the untreated samples. Quintero-Lira et al. [22] showed that all types of honey subjected to ultrasound for 5, 10, and 15 min showed significant increases in antioxidant activity, mentioning that this type of processing is highly effective for the extraction of bioactive compounds with antioxidant activity. On the other hand, Ruiz-Navajas et al. [16] determined the antioxidant activity by DPPH in various samples of Mexican honey, finding that the values differed widely between the samples, and attributing this phenomenon to the presence of phenolic compounds and flavonoids; they mentioned that the activity of the extracts containing bioactive compounds is related to the scavenging of free radicals, the donation of hydrogen, the chelation of metal ions, or even as a substrate for superoxide or hydroxyl radicals, in addition to a variety of factors such as concentration, temperature, light, type of substrate, and the physical state of the system.

### 3.4. Enzyme Inhibition

Ali et al. [24] determined the activity of honeys with different botanical origins by varying the concentration of the sample, finding behavior similar to that observed in the present work, since all their samples presented the inhibition of α-amylase, and an inhibition level directly proportional to the concentration of honey. They found the greatest activity in tualang honey, attributing the inhibition to the phenolic compounds present in this honey, such as kaempferol, caffeic acid, and p-coumaric acid. Devarajan and Venugopal [8] evaluated the inhibition of α-amylase with honey extracts from India, finding that the inhibition of the extracts was dependent on the concentration used, attributed to flavonoids such as luteolin, myricetin, and quercetin, which suggests that these compounds can act as possible inhibitors against the enzyme. They suggested that the extracts could become an alternative for the inhibition of the breakdown of carbohydrates and the control of the glycemic index of food products, in addition to other effects, such as antimicrobial, which enhance the honey’s potential therapeutic activity. The results obtained for enzyme inhibition may be related to the increase in bioactive compounds described above, due to these studies indicating a direct relationship between enzyme inhibition and the increase in bioactive compounds, and demonstrating that, with ultrasound, the benefits of honey can be potentiated.

Ali et al. [24], in their antidiabetic study of various honey samples, evaluated the inhibitory activity of α-glucosidase, finding that all their samples had an effect, proportional to the concentration, achieving up to a maximum of 68.32%; this behavior was attributed to the botanical origin of the honey, specifically from phenols and flavonoids. Ali Asgar [25] mentioned that phenolic compounds can have a potential antidiabetic effect, specifically referring to tannins and flavonoids, among others. Zhang et al. [26] identified some phenolic acids, flavonoids, and other pigments from raspberries and observed that these compounds have a strong inhibitory activity.

Ultrasound has proven to be an effective aid for the potentiation of bioactive compounds related to the inhibition of α-glucosidase. Li et al. [27] evaluated the effect of ultrasound extraction of bioactive compounds from longan fruit peel and determined their inhibitory effects against α-glucosidase, finding that the inhibition is proportional to the amount of extract, and mentioning that this is because the ultrasound process significantly improves the extraction; by increasing these components, inhibition is favored, which translates into a reduction in the release of monosaccharides after consumption.

### 3.5. In Vitro Digestibility

O’Sullivan et al. [28] evaluated the effect of in vitro digestion on commercial honeys, mentioning that polyphenols and other bioactive compounds are highly susceptible to degradation during digestion, due to both the pH and the enzymes present. Cianciosi et al. [29] mentioned that the mechanism of the hydrolysis, enzymatic or bacterial, of flavonoids leads to the glycosylated form of these compounds, and it is necessary to consider the conditions of digestion, such as the enzymatic content, since this depends on the greater or lesser transformation of the flavonoids, directly influencing their bioavailability. Zafra-Rojas et al. [30] mentioned that the use of ultrasound helps in the extraction of phenolic compounds and can also improve bioaccessibility.

The decrease in antioxidant activity after digestion depends on many factors, according to O’Sullivan et al. [28]: specifically on the matrix that contains them, due to the physicochemical properties and the interactions between components. The decrease in antioxidant capacity can be attributed to the decrease in the content of polyphenols or other bioactive compounds in the extracts after digestion, or to the change in the polarity of the gastrointestinal digestion, which makes it more difficult to react with free radicals [31]. Zafra-Rojas et al. [30] indicated that the application of ultrasound and subsequent hydrolysis can lead to the high extraction of individual phenolic compounds compared to non-ultrasonicated samples, indicating that, despite the decrease during digestibility, there are still compounds present that can positively impact health. Cianciosi et al. [29] mentioned that, during the gastrointestinal digestion of honey, various compounds with antioxidant activity are released from the food matrix, which not only belong to phenolic compounds, but also to proteins, vitamins, and organic acids, thus being the reason for the determination of antioxidant activity by more than one method. However, these authors mentioned that, due to the nature of the compounds with this activity, there will be some that are more stable at specific pH conditions or some other factors, such as those mentioned above.

Parada et al. [32] determined that the phenolic compounds from honey reduce the in vitro digestibility of starch, finding a direct relationship between the inhibition of α-amylase and α-glucosidase and the concentration of phenolic compounds, and pointing out the ability of these compounds to inhibit enzymes related to the degradation of carbohydrates. There are reports of the use of bioactive compounds from other food sources to inhibit enzymes related to carbohydrate degradation. Medina-Pérez et al. [7] used bioactive compounds from acid fruits of *Opuntia*, reporting the presence of phenols and flavonoids mainly, to evaluate their inhibition of α-amylase and α-glucosidase. After subjecting them to in vitro digestion, they found that the bioactive compounds of this fruit that remained after the treatment generated a maximum inhibition greater than 60% for α-amylase, while for α-glucosidase, they reported a maximum inhibition greater than 40%. Their results suggest that this fruit is an effective alternative for postprandial glycemic control and exerts antidiabetic effects.

## 4. Materials and Methods

### 4.1. Materials

The materials used in the present study were ethanol (HPLC grade), ascorbic acid (Sigma-Aldrich, U.S.A.), gallic acid (J.T. Baker, Phillipsburg, NJ, U.S.A.), quercetin (Sigma-Aldrich, St. Louis, MO, U.S.A.), sodium bicarbonate (Sigma-Aldrich, St. Louis, MO, U.S.A.), Folin–Ciocalteau reagent (Sigma-Aldrich, St. Louis, MO, U.S.A.), sodium carbonate (J.T. Baker, Phillipsburg, NJ, U.S.A.), aluminum trichloride (Sigma-Aldrich, St. Louis, MO, U.S.A.), sodium hydroxide (J.T. Baker, Phillipsburg, NJ, U.S.A.), 2,2-diphenyl-1-picrylhydrazyl (DPPH, Sigma-Aldrich, St. Louis, MO, U.S.A.), 2, 2-Azino-bis (acid 3 -ethylbenzothiazoline-6-sulfonic) (ABTS, Sigma-Aldrich, St. Louis, MO, U.S.A.), potassium persulfate (analytical reagent grade), α-amylase (Sigma-Aldrich, St. Louis, MO, U.S.A.), α-glucosidase (Sigma-Aldrich, St. Louis, MO, U.S.A.), pepsin (Sigma-Aldrich, St. Louis, MO, U.S.A.), pancreatin (Sigma-Aldrich, St. Louis, MO, U.S.A.), bile salts (Sigma-Aldrich, St. Louis, MO, U.S.A.), dinitrosalicylic acid (Panreac, Barcelona, Spain), dextrose (Sigma-Aldrich, St. Louis, MO, U.S.A.), and 4-Nitrophenyl-α-D-glucopyranoside (Sigma-Aldrich, St. Louis, MO, U.S.A.). Acarbose (Sigma-Aldrich, St. Louis, MO, U.S.A.), hydrochloric acid (J.T. Baker, Phillipsburg, NJ, U.S.A.), and sodium phosphate buffer (Sigma-Aldrich, St. Louis, MO, U.S.A.) were also used.

### 4.2. Honey Samples

The honeys evaluated were obtained from three different municipalities in the state of Oaxaca, Mexico (17°7′0″ N, 97°40′0″ E), with a total of 6 samples (Table 4), which were coded with abbreviations according to the site where they were obtained. The containers were hermetically sealed. Subsequently, the samples were taken to the laboratory of the Instituto de Ciencias Agropecuarias of the Universidad Autónoma del Estado de Hidalgo for analysis, and stored at 15 °C in complete darkness [6]. For all the variables analyzed, each treatment was evaluated in triplicate, and, in order to ensure repeatability, all the analyses described below were performed twice.

### 4.3. Melissopalynological Analysis

The pollen analysis was developed with respect to the methodology described by Pospiech et al. [33], with some modifications. A sample of 10 g was mixed with 40 mL of distilled water at a temperature of 40 °C. The sample was filtered using a vacuum; subsequently, it was dried (24 h). The resultant sample containing the pollen grains was placed on a slide and covered with a coverslip. After drying, the edges of the coverslip were covered with a layer of varnish. The samples were observed under an Olympus CX31 microscope (Olympus corporation, Tokyo, Japan) coupled to an Infinity 1-2C camera and were analyzed using Image-Pro Plus software (Media Cybernetics, Silver Spring, MD, USA). Pollen grains were captured in five different focal planes at distances of 8 µm from each other, and a depth-of-focus image was created, which was later used to determine the botanical species of the pollen grains. Microscope slides were positioned so that at least 300 grains of pollen were captured, and these were subsequently analyzed.

### 4.4. Ultrasonic Processing

The extracts of the different honeys were obtained according to Peláez-Acero et al. [12], with some modifications. First, 5 g of honey in 85% ethanol (1:10) was placed in an ultrasonic bath, the Branson 3510 (Branson, CT, USA), with a 400-W high-intensity device at a frequency of 42 kHz, with a 50% duty cycle, for 10, 20, and 30 min at a constant temperature (20 °C). Samples were homogenized and centrifuged at 6000 rpm for 15 min at 4 °C, and the supernatant was saved for subsequent analysis.

### 4.5. Determination of Total Phenols

The determination of total phenols was carried out according to the Folin–Ciocalteu method, as described by Peláez-Acero et al. [12]. Samples were diluted (1:10) using distilled water, homogenized, and centrifuged at 10,000 rpm for 15 min at 4 °C. Subsequently, 0.3 mL of the supernatant was taken and mixed with 1.5 mL of Folin–Ciocalteu 0.2 N reagent for 8 min; then, 1.2 mL of 0.7 M Na_2_CO_3_ was added, and the mixture was left to react for two hours in darkness. The resulting mixture was measured at 765 nm using a Jenway 6715 UV–Vis spectrophotometer (Jenway, Staffordshire, UK). The total phenol content was determined from a gallic acid curve. The amount of total phenols was expressed as gallic acid equivalents/100g of honey (mg GAE/100 g).

### 4.6. Determination of Total Flavonoids

The total content of flavonoids was determined using the method described by Peláez-Acero et al. [12]. First, 5 mL of the extract was added to 5 mL of pure methanol, homogenized, and centrifuged at 10,000 rpm for 15 min. Then, 2 mL of the supernatant was placed in a test tube and 2 mL of 2% AlCl_3_ was added, and the mixture was kept in the dark for 20 min. The absorbance at 415 nm was measured with a UV–Vis spectrophotometer. Quercetin was used for the standard curve. The total content of flavonoids was expressed in mg quercetin equivalent/100 g of honey (mg QE/100 g).

### 4.7. Antioxidant Activity

#### 4.7.1. 2,2′-Azino-Bis-3-Ethylbenzothiazoline 6-Sulfonic Acid (ABTS)

The antioxidant activity was determined using the ABTS radical method, according to Quintero-Lira et al. [22]. First, 7 μM ABTS reagent was reacted with 2.45 μM potassium persulfate (K_2_S_2_O_8_) in a 1:1 ratio; the reaction was left under stirring for 16 h in darkness. The radical was stabilized with 20% ethanol until it reached a value in the UV–Vis spectrophotometer of 0.70 ± 0.01 at 734 nm. Once the reagent was stabilized, 2 mL was placed in a tube and 200 μL of the honey extract was added; the mixture was stirred and left to stand for 6 min, and then the absorbance was read. The results were expressed as ascorbic acid equivalents/100 g of honey (mg ESA/100 g).

#### 4.7.2. 2,2-Diphenyl-1-Picrylhydrazyl (DPPH)

The DPPH radical scavenging activity was assessed according to Quintero-Lira et al. [22]. For the preparation of the DPPH, 7.9 mg was weighed and dissolved in 100 mL of 80% methanol, placed under stirring for 2 h in total darkness for total dissolution, stabilized with 80% methanol until it reached an absorbance of 0.70 ± 0.01 at 515 nm, and 2.5 mL of DPPH and 500 μL of honey extract were added. Subsequently, it was left to stand for 1 h in total darkness, and the absorbance at 515 nm was measured with a UV–Vis spectrophotometer. The results were expressed as ascorbic acid equivalents/100 g of honey (mg ESA/100 g).

### 4.8. Antidiabetic Activity

#### 4.8.1. Inhibition of α-amylase

The determination of the inhibition of α-amylase in the honeys was performed according to Medina-Pérez et al. [7], with modifications. First, 100 µL of the sample was mixed with 100 µL of 0.02 mol/L sodium phosphate buffer (pH 6.9) and 100 µL of α-amylase buffer solution (1 U/mL), and pre-incubated at 37 °C for 10 min. After this time, 100 µL of aqueous starch solution (0.1%) was added and the mixture incubated at 37 °C for 60 min. The reaction was stopped with 1 mL of dinitrosalicylic acid reagent. Samples were incubated in a 90 °C water bath for 5 min and immediately cooled in an ice bath. Acarbose was used as a positive control for this assay; a negative control without inhibitory compounds was established, adding distilled water.

#### 4.8.2. Inhibition of α-glucosidase

The inhibition of α-glucosidase was assessed following the methodology developed by Medina-Pérez et al. [7], with modifications. First, 10 µL of sample was mixed with 100 µL of sodium phosphate buffer (0.05 mol/L) at pH 6.9, and 0.25 µL of glucosidase solution (25 mg/mL) was pre-incubated at 37 °C for 10 min. Then, 25 µL of 3 mM 4-nitrophenyl α-D-glucopyranoside (pNPG) substrate was added and incubated at 37 °C for 40 min. The reaction was stopped by the addition of 25 µL of sodium carbonate (0.1 M), and the absorbance at 405 nm was measured. The results were expressed as a percentage of inhibition. Acarbose was used as a positive control for this assay; a negative control without inhibitory compounds was established, adding distilled water.

### 4.9. In Vitro Digestion

#### 4.9.1. Gastric Phase

The gastric phase was carried out in accordance with the method reported by Medina-Pérez et al. [7], with some modifications. In this phase, 5 mL of honey extract was combined with 20 mL of gastric juice at pH 6 (HCl 6 N) (40,000 u pepsin, CaCl_2_ 0.3 M in HCl 0.1 M), and then incubated in a water bath with shaking for 120 min at 37 °C, after which 12.5 mL was kept. The retained mixture was centrifuged at 6000 rpm for 15 min at 4 °C for further analysis (total phenols, total flavonoids, ABTS, DPPH, and α-amylase and α-glucosidase inhibition), and the rest proceeded to the next step.

#### 4.9.2. Intestinal Phase

The intestinal phase was carried out in accordance with the method reported by Medina-Pérez et al. [7]. The supernatant obtained from the gastric phase was combined, adjusting the pH to 7, with 0.5 M NaHCO_3_, and 1.25 mL of pancreatin-bile mixture (0.4 g pancreatin, 2.5 g bile salts in 100 mL of 0.1 M NaHCO_3_) was later added. Then, the mixture was incubated in a water bath with shaking for 120 min at 37 °C; this mixture was centrifuged at 6000 rpm for 15 min at 4 °C, and the supernatant was used for the respective analyses (total phenols, total flavonoids, ABTS, DPPH, α-glucosidase inhibition, and α-amylase inhibition).

### 4.10. Statistical Analysis

For the statistical analysis, a completely randomized design was used. The results were interpreted with an analysis of variance, and, when significant differences were observed between the different processes in the different honeys (*p* < 0.05), the comparison of means was performed by Tukey’s method using the NCSS2007 software (USA).

## 5. Conclusions

In the present work, it is shown that both the honey and the ultrasound time have a significant impact on the properties of Mexican honeys. It should be noted that each type of honey is unique and has specific characteristics, including its composition, climate, and harvest area, among others; the behavior of each honey depends on these factors. After the ultrasound treatment, the bioactive compounds and antioxidant activity, as well as enzyme inhibition, showed a significant increase. This increase was more notable in P-A1 and A-B, with 20 min being the best sonication time. However, the properties of the honeys were affected when they were subjected to in vitro digestion, observing a decrease after the gastrointestinal phase; therefore, more research is required in order to be able to better preserve these compounds after digestion. The results of this work provide interesting information to further the research on new technologies and sources that work as auxiliaries in the treatment of various diseases, such as diabetes, using ingredients that are used in traditional medicine in many cultures.

## Figures and Tables

**Table 1:** Melissopalynological analysis of the honeys.

Code	Municipality	Botanical Origin
J-A	San Jeronimo Tecoalt	*Macroptilium* (21.30 ± 1.50%),
		*Trema micrantha* (16.00 ± 2.10%),
		*Cyperaceae* (14.3 ± 0.05%),
		*Quercus* sp. (10.70 ± 2.10%),
		*Lonchocarpus* sp. (9.15 ± 1.20%),
		*Cochlospermum vitifolium* (7.00 ± 0.00%).
J-A1	San Jeronimo Tecoalt	*Trema micrantha* (13.70 ± 0.10%),
		*Ambrosia tenuifolia* (10.0 ± 0.00%),
		*Psidium* sp. (7.0 ± 0.00%),
		*Cyperaceae* (7.0 ± 0.00%),
		*Vitis tiliifolia* (7.50 ± 0.05%),
		*Lonchocarpus* sp. (6.30 ± 0.20%).
P-A	San Pedro Ocopetatillo	*Vitis tiliifolia* (12.7 ± 0.30%),
		*Viguiera dentata* (12.3 ± 0.01%),
		*Melothria pendula* (11.3 ± 0.50%),
		*Montanoa grandiflora* (7.7 ± 0.00%),
		*Melicoccus oviliformis* (7.3 ± 0.50%),
		*Pentacalia ledifolia* (6.7 ± 0.00%).
P-B	San Pedro Ocopetatillo	*Brassica campestris* (35.70 ± 0.20%),
		*Vitis tiliifolia* (11.7 ± 0.20%),
		*Eucalyptus ficifolia* (10.0 ± 1.10%),
		*Trema micrantha* (8.3 ± 0.50%),
		*Melicoccus oviliformis* (5.7 ± 0.10%),
		*Ambrosia tenuifolia* (4.7 ± 0.10%).
P-A1	San Pedro Ocopetatillo	*Vitis tiliifolia* (23.3 ± 0.00%),
		*Melicoccus oviliformis* (14.0 ± 0.20%),
		*Litsea* sp. (8.0 ± 0.50%),
		*Melothria pendula* (7.3 ± 0.10%),
		*Ageratum conyzoides* (5.7 ± 0.50%),
		*Trema micrantha* (4.7 ± 0.00%).
A-B	San Antonio Eloxochitlán De Flores Magón	*Ambrosia tenuifolia* (22.3 ± 1.20%),
		*Lonchocarpus* sp. (10.7 ± 0.00%),
		*Trema micrantha* (7.3 ± 0.30%),
		*Brassica campestris* (6.7 ± 0.20%),
		*Vitis tiliifolia* (5.7 ± 0.50%),
		*Cyperaceae* (5.3 ± 0.00%).

**Table 2:** In vitro digestibility of bioactive compounds and antioxidant activity at 20 min of ultrasound.

	J-A	J-A1	P-A	P-B	P-A1	A-B
**Total phenols** (mg GAE/100 g)						
Initial	18.72 ± 0.19 ^Ac^	22.18 ± 1.17 ^Ab^	23.56 ± 4.69 ^Ab^	28.26 ± 1.56 ^Aa^	29.91 ± 1.95 ^Aa^	26.87 ± 1.56 ^Aa^
Gastric	4.06 ± 0.07 ^Ba^	3.79 ± 0.02 ^Bb^	3.89 ± 0.05 ^Bb^	3.62 ± 0.04 ^Bb^	3.81 ± 0.05 ^Bb^	4.40 ± 0.02 ^Ba^
Intestinal	3.98 ± 0.40 ^Ba^	3.58 ± 0.05 ^Ba^	3.62 ± 0.36 ^Ba^	3.57 ± 0.04 ^Ba^	3.76 ± 0.71 ^Ba^	4.07 ± 0.02 ^Ba^
**Total flavonoids** (mg QE/100 g)						
Initial	0.86 ± 0.01 ^Ab^	0.71 ± 0.09 ^Ac^	0.63 ± 0.01 ^Ad^	1.93 ± 0.01 ^Aa^	0.88 ± 0.01 ^Ab^	0.97 ± 0.06 ^Ab^
Gastric	0.48 ± 0.04 ^Bd^	0.60 ± 0.03 ^Bc^	0.10 ± 0.01 ^Bf^	1.41 ± 0.03 ^Ba^	0.23 ± 0.01 ^Be^	0.86 ± 0.09 ^ABb^
Intestinal	0.44 ± 0.02 ^Bd^	0.57 ± 0.02 ^Bc^	0.07 ± 0.01 ^Bf^	1.26 ± 0.03 ^Ca^	0.19 ± 0.04 ^Be^	0.80 ± 0.04 ^Bb^
**ABTS** (mg AAE/100 g)						
Initial	13.81 ± 1.22 ^Ae^	27.78 ± 0.30 ^Aa^	17.25 ± 3.04 ^Acd^	16.17 ± 0.91 ^Ad^	22.62 ± 0.30 ^Ab^	20.26 ± 0.61 ^Ac^
Gastric	10.41 ± 1.69 ^Be^	21.81 ± 1.35 ^Ba^	13.03 ± 0.68 ^Bd^	15.61 ± 0.68 ^Bc^	19.19 ± 0.34 ^Bb^	12.51 ± 0.34 ^Bd^
Intestinal	7.30 ± 0.68 ^Cd^	8.74 ± 0.35 ^Cc^	8.26 ± 0.68 ^Ccd^	10.41 ± 0.34 ^Ca^	9.69 ± 0.01 ^Cb^	8.97 ± 0.34 ^Cc^
**DPPH** (mg AAE/100 g)						
Initial	17.23 ± 0.01 ^Aab^	19.51 ± 2.51 ^Ab^	19.97 ± 1.29 ^Ab^	18.60 ± 3.51 ^Ab^	17.23 ± 3.87 ^Ab^	36.38 ± 3.87 ^Aa^
Gastric	16.70 ± 3.62 ^Aabc^	18.24 ± 1.45 ^Aa^	16.70 ± 0.72 ^Bbc^	17.73 ± 2.17 ^Aa^	14.65 ± 2.17 ^Ac^	19.77 ± 0.72 ^Ba^
Intestinal	10.55 ± 2.17 ^Bbc^	7.99 ± 1.45 ^Bc^	13.11±1.45 ^Cab^	15.16 ± 1.45 ^Aa^	10.55 ± 2.17 ^Bbc^	13.11 ± 1.45 ^Cab^

The results are expressed as means ± standard deviation. Capital letters indicate significant differences (*p* < 0.05) in the same sample of honey (columns). Lowercase letters indicate significant differences (*p* < 0.05) between the samples for each stage of digestibility (rows).

**Table 3:** Effect of in vitro digestibility on enzyme activity.

	α-amylase (% Inhibition)			α-glucosidase (% Inhibition)		
	Initial	Gastric	Intestinal	Initial	Gastric	Intestinal
Control +	61.55 ± 0.11 ^Ca^	69.77 ± 1.08 ^Ba^	88.47 ± 1.18 ^Aa^	69.55 ± 0.11 ^Ca^	81.22 ± 1.08 ^Ba^	89.22 ± 1.18 ^Aa^
Control −	0.21 ± 0.01 ^Ag^	0.19 ± 0.01 ^Af^	0.19 ± 0.02 ^Af^	0.22 ± 0.01 ^Ag^	0.20 ± 0.03 ^Ag^	0.19 ± 0.01 ^Af^
J-A	19.49 ± 0.03 ^Ad^	9.03 ± 1.42 ^Be^	8.14 ± 3.34 ^Bd^	11.60 ± 0.03 ^Ae^	8.91 ± 0.18 ^Be^	7.05 ± 0.01 ^Cc^
J-A1	15.16 ± 0.73 ^Af^	12.42 ± 0.21 ^Bd^	2.12 ± 2.17 ^Ce^	16.85 ± 0.80 ^Ad^	10.93 ± 2.17 ^Bcd^	7.54 ± 0.35 ^Cc^
P-A	17.68 ± 0.32 ^Ae^	12.33 ± 0.08 ^Bd^	10.09 ± 0.25 ^Cc^	9.62 ± 0.35 ^Af^	7.42 ± 0.35 ^Bf^	6.55 ± 0.44 ^Bd^
P-B	26.87 ± 0.76 ^Ac^	15.40 ± 0.42 ^Bc^	14.13 ± 2.29 ^Bb^	19.71 ± 0.83 ^Ac^	10.65 ± 0.44 ^Bd^	5.28 ± 0.22 ^Ce^
P-A1	37.15 ± 0.09 ^Ab^	19.56 ± 0.29 ^Bb^	9.53 ± 0.04 ^Cc^	30.99 ± 0.10 ^Ab^	12.57 ± 0.70 ^Bc^	9.75 ± 0.04 ^Cb^
A-B	28.54 ± 0.09 ^Ac^	15.22 ± 0.17 ^Bc^	13.54 ± 0.21 ^Bb^	21.54 ± 0.10 ^Ac^	14.50 ± 0.18 ^Bb^	9.62 ± 0.22 ^Cb^

The results are expressed as means ± standard deviation. Capital letters indicate significant differences (*p* < 0.05) in the same sample of honey (rows). Lowercase letters indicate significant differences (*p* < 0.05) between the samples for each stage of digestibility (columns). Control +: acarbose, Control −: distilled water.

**Table 4:** Honey coding.

Municipality	Color	Code
San Jerónimo Tecoalt	Amber	J-A
San Jerónimo Tecoalt	Amber	J-A1
San Pablo Ocopetatillo	Amber	P-A
San Pablo Ocopetatillo	White	P-B
San Pablo Ocopetatillo	Amber	P-A1
San Antonio Eloxochitlán	White	A-B

## Data Availability

Not applicable.
